# The Tight Relationship Between the Tumoral Microenvironment and Radium-223

**DOI:** 10.3390/biomedicines13020456

**Published:** 2025-02-12

**Authors:** Miriam Conte, Miriam Tomaciello, Maria Silvia De Feo, Viviana Frantellizzi, Francesco Marampon, Flaminia De Cristofaro, Giuseppe De Vincentis, Luca Filippi

**Affiliations:** 1 Department of Radiological Sciences, Oncology and Anatomical Pathology, Sapienza, “Sapienza” University of Rome, 00161 Rome, Italy; miriam.conte@uniroma1.it (M.C.); miriam.tomaciello@uniroma1.it (M.T.); mariasilvia.defeo@uniroma1.it (M.S.D.F.); viviana.frantellizzi@uniroma1.it (V.F.); francesco.marampon@uniroma1.it (F.M.); flaminia.decristofaro@uniroma1.it (F.D.C.); giuseppe.devincentis@uniroma1.it (G.D.V.); 2 Department of Biomedicine and Prevention, University of Rome “Tor Vergata”, Via Montpellier 1, 00133 Rome, Italy

**Keywords:** alpha-emitter, radium-223, tumor microenvironment, PARP, chemotherapy, prostate cancer, hormonal therapy, abiraterone, *BRCA*, osteoblasts

## Abstract

Radium-223 (^223^Ra) was the first radioactive isotope approved for treating castration-resistant prostate cancer (CRPC) with symptomatic bone metastases without visceral metastatic disease. To better understand the action of ^223^Ra, its role in the tumor microenvironment represents a crucial aspect. A literature search was conducted using the PubMed/MEDLINE database and studies regarding the relationship between ^223^Ra and the tumoral microenvironment were considered. The tumoral microenvironment is a complex setting in which complex interactions between cells and molecules occur. Radium-223, as an alpha-emitter, induces double-stranded DNA breaks; to potentiate this effect, it could be used in patients with genetic instability but also in combination with therapies which inhibit DNA repair, modulate the immune response, or control tumor growth. In conclusion, a few studies have taken into consideration the tumoral microenvironment in association with ^223^Ra. However, its understanding is a priority to better comprehend how to effectively exploit ^223^Ra and its action mechanism.

## 1. Introduction

According to the global cancer observatory [1], prostate cancer (PCa) was the neoplasm with the highest incidence among men in 2022 after lung cancer (1,467,854 vs. 1,572,045) but had a lower mortality compared to lung, liver, colorectum and stomach cancers. The majority of cases are diagnosed at an early stage and often have an indolent course. However, it is estimated that up to one-third of prostate cancer patients develop metastases [2]. The basis of metastatic hormone-sensitive prostate cancer (mHSPC) treatment is achieving testosterone castration levels through bilateral orchidectomies or by medical castration through androgen deprivation therapy (ADT), such as the use of gonadotropin-releasing hormone (GnRH) analogues.

Castration-resistant prostate cancer (CRPC) is the condition in which prostate cancer clinically, radiographically or biochemically progresses despite castration levels of serum testosterone (<50 ng/dL; <1.7 nmoL/L) [3]. Radiographic progression is defined as the appearance of at least two new lesions on bone scan scintigraphy or as the progression of a measurable lesion according to the RECIST method. Biological progression is defined as three consecutive increases in the PSA (prostate-specific antigen) level at least one week apart, with two rises of at least 50% from the lowest PSA hematic level and a PSA level above 2 ng/mL [4]. The mechanism of development of CRPC is not completely clear. Two main theories have been proposed: the androgen receptor (AR) pathway-dependent and independent theories. Point mutations in AR cause increased sensitivity of the receptor, which can then be stimulated not only by androgens, but also by estrogen and progesterone, which can activate androgen-independent gene transcription. Truncated AR isoforms lead to the constitutive activation of AR on tumor cells, making them independent of androgen proliferative stimuli. Another circumstance is a reduction in AR corepressors and an augmentation of AR coactivators, consequently leading to the overactivation of ARs. The androgen-independent pathway involves mitogen-activated protein kinase (MAPK), phosphoinositide 3-kinase (PI3K) and phosphoenolpyruvate carboxykinase (PCK). These enzymes activate alternative pathways for tumoral cell growth and survival which are independent of AR signaling [5]. A GnRH agonist, which suppresses luteinizing hormone (LH) production and thus testicular androgen synthesis, is the first-line treatment. Unfortunately, ADT-treated mHSPC often progresses to a metastatic castration-resistant state (mCRPC), characterized by castration serum testosterone levels (less than 50 ng/dL), which are associated with either biochemical or radiological progression [6]. A commonly used strategy to prolong the hormone-sensitive phase (often called maximal androgen blockade) is adding first-generation anti-androgens such as bicalutamide to ADT. While ADT remains the mainstay of treatment, the addition of docetaxel chemotherapy and second-generation anti-androgens (such as abiraterone, apalutamide and enzalutamide) to androgen deprivation therapy has shown a survival benefit [7]. As previously stated, this treatment unfortunately selects for cancer cells that will develop resistance to androgen deprivation, leading to CRPC [5]. However, ADT with an LHRH agonist or antagonist must be continued to maintain serum castration testosterone levels in patients who develop CRPC [3]. The castration condition can be achieved using a variety of drugs, including LHRH agonists and LHRH antagonists, luteinizing hormone-releasing hormone (LHRH) agonists, also known as LHRH analogues, or gonadotropin-releasing hormone (GnRH) agonists, including leuprolide, goserelin, triptorelin and histrelin. Degarelix is an LHRH antagonist approved for the treatment of PCa [8]. Currently, there is a strong recommendation to combine ADT with androgen synthesis inhibitors (e.g., abiraterone acetate) or anti-androgen therapies (e.g., enzalutamide, apalutamide, or darolutamide), as well as with chemotherapy (docetaxel) [9]. Several chemotherapeutic agents have been approved for the treatment of PCa, including taxanes (docetaxel, cabazitaxel), mitoxantrone and estramustine. Other chemotherapeutic agents, including cisplatin, oxaliplatin and carboplatin, are currently undergoing clinical trials to evaluate their use in PCa [10]. Docetaxel is used as the first-line chemotherapy and cabazitaxel as the second-line treatment to overcome docetaxel resistance [11]. Immunotherapy aims to boost the patient’s immune system to fight cancer cells and represents another important weapon against cancer. There are currently three immunotherapy drugs with Food and Drug Administration (FDA) approval for the treatment of CRPC [12]. Prostate cancer cells express several proteins, including PSA and prostatic acid phosphatase (PAP), which could be targeted by immunotherapy [13]. Sipuleucel-T was the first immunotherapy with FDA approval. Subsequently, pembrolizumab and dostarlimab were approved by the FDA for advanced solid tumors including CRPC. In particular, pembrolizumab and dostarlimab were approved for patients with unresectable or metastatic microsatellite instability-high or mismatch repair-deficient (dMMR) tumors [12,14]. Immunotherapies such as sipuleucel-T and immune checkpoint inhibitors (ICIs) offer an attractive alternative to standard treatments for CRPC such as chemotherapy and hormone therapy. However, unlike other solid tumors, there is no evidence of an optimal response in CRPC [5]. Painful bone metastases significantly affect the quality of life of mCRPC patients. Bisphosphonates, which are widely used inhibitors of bone resorption, were the first bone-targeting drugs [15]. Another bone-targeted treatment for symptomatic skeletal metastases is the radiopharmaceutical radium-223 (^223^Ra, ^223^RaCl_2_), which provides a survival benefit and symptomatic relief [16]. Radium-223 is approved for use in adult patients with CRPC and symptomatic bone metastases with no known visceral metastases and lymphadenopathies smaller than 3 cm [17]. Recent guidelines do not establish the safety and efficacy of concomitant chemotherapies with ^223^Ra but further studies are discussed in the section below. Approximately 20–30% of patients with prostate cancer will experience bone recurrences that result in pain, pathological fracture, spinal cord compression, which may reduce the patients’ quality of life. Since radium-223 is a α-emitting radionuclide that is absorbed into the bone matrix in active mineralization sites via osteoblasts, similar to calcium [18]. The tumoral microenvironment represents a crucial aspect to highlight; therefore, the molecular aspects of the action of ^223^Ra in the microenvironment will be analyzed in Section 5. It is well known that prostate cancer cells (PCs) play a role in preparing the bone for metastasis [19,20,21] through the production of growth factors, chemokines and cytokines that stimulate vascularization and facilitate the homing of PCs themselves, which are also supported by white adipose tissue and the bone marrow. PCs stimulate osteoblasts, which can activate osteoclasts through nuclear factor (NF-kappaB) ligand (RANKL) signaling in order to create more space for the growth of osteoblastic lesions [22]. The higher level of osteoprotegerin, an osteoblastic protein that inhibits osteoclasts, in patients with PCa also suggests a role of osteoblasts in bone cancer progression [23]. The aim of this review is to focus on the relationship between PCs, the tumor microenvironment, and radium-223 to highlight the salient aspects of radium’s action in bone metastases.

## 2. Radium-223: Definitions and Therapeutic Effect

Radium-223 was the first alpha-emitter radioactive isotope approved for treating castration-resistant prostate cancer (CRPC) with symptomatic bone metastases without visceral metastatic disease [17]. During its decay, this isotope emits one alpha particle and other three alpha particles through a multistep chain of progeny, which are responsible for the therapeutic effect of radium. An alpha particle (α-particle) consists of a helium-4 nucleus (4He), formed by two protons and two neutrons, and has significantly higher Linear Energy Transfer (LET) than beta particles, ranging from 50 to 230 keV/µm, with a mean energy deposition of 100 keV/µm. This high LET results in a short range of penetration of α-particles into matter, approximately 5–10 cell diameters (28–100 µm), ensuring that the radiation primarily affects the targeted cells and the closely neighboring cells while sparing more healthy cells, as demonstrated by the low bone marrow toxicity [24,25]. α-particles induce double-stranded breaks (DSBs) in DNA, and this damage occurs independently of tissue oxygenation and of cellular resistance, which are visible in other therapies such as photon irradiation and chemotherapy [26]. As previously stated, as a calcium analogue, 25% of the radium is taken up by bones in areas with active osteoblasts, which can be visualized using a gamma camera thanks to its gamma emissions [27]. Both the passive binding of radium-223 as a calcium mimic to hydroxyapatite and the active incorporation by osteoblasts have been shown to contribute to the binding of radium-223 to bone tissue [28]. Moreover, radium inhibits the differentiation of osteoclasts through its alpha particle energy, which causes cellular disruption, and by inhibiting osteoclasts’ RANKL secretion, preserving the normal bone matrix [29]. Radium-223 also appears to activate the immune system, inhibit immune suppression and alter the tumor cell phenotype so that it is more susceptible to immune-mediated cell death [30,31]. This appears to be related to the activation of the endoplasmic reticulum (ER) stress response pathway involving the transmembrane protein kinase-like endoplasmic reticulum kinase (PERK), which mediates the unfolded protein response, resulting in increased expression of several components of the antigen-processing machinery [32]. Radium can also stimulate the immune response through the activation of the stimulator of interferon genes (*STING*) pathway [33,34]. *STING* is an ER translocon-associated transmembrane protein that detects pathogenic DNA or damaged host DNA (due to apoptosis or necrosis) in the cytosol and triggers innate immune responses [34]. How radium-223 activates the *STING* pathway is not yet known. The biological response after ^223^Ra therapy is assessed using the decrease in alkaline phosphatase (ALP) levels [18,35], even though there are no adequate biomarkers defined for patient selection and therapeutic response evaluation. Recently, bone ALP levels were demonstrated to be a prognostic factor in patients treated with radium, which could predict which patients have a higher probability to survive for 2 years and identify who could potentially benefit from earlier treatment with radium and delaying conventional chemotherapy. In fact, low baseline levels of bone ALP were associated with >20% higher 2-yr survival rates [36,37,38,39]. The efficacy of earlier radium-223 therapy was also suggested by the study of Casarin et al. [40]. The authors developed a silico model of bone metastatic PCa that is capable of identifying micro-lesions close to the bone interface, which were the best targets regarding the regression/eradication results. Conversely, one quarter of patients treated with ^223^Ra that experienced vertebral fractures had a decrease in serum levels of ALP. Thus, this reduction seems to be correlated with the likelihood of developing a vertebral fracture during radium therapy. Some authors explained this phenomenon based on the capacity of ^223^Ra to inhibit osteoblasts and therefore to induce bone fragility [41]. This could lead to bone remodeling in the case of enhanced osteoclast activity, as demonstrated by the increased incidence of vertebral fracture in patients with previous vertebral fractures [42,43]; therefore, a sort of “domino effect” occurs in patients who previously had osteoporosis and were treated with radium-223 [44]. Since the decrease in ALP levels is associated with the development of vertebral fractures, this suggests that radium-223 treatment is a risk factor for vertebral fractures in patients with concomitant bone fragility due to androgen deprivation therapy [45,46].

Regarding prostate-specific antigen (PSA), during some therapies such as luteinizing hormone-releasing hormone (LHRH) agonist treatment and systemic chemotherapy, especially using cabazitaxel and docetaxel, the so-called flare phenomenon may occur. It is an early and transient rise in the PSA level followed by a decrease of at least 50% from the baseline or from the PSA peak value. An undefined PSA response (though it does not have a clear definition) also affects the extent of the following decline [47,48,49,50,51]. A PSA flare is very common in patients undergoing radium treatment, as demonstrated by De Vincentis et al. in their case of a patient with a rise in PSA after the fifth cycle of radium without presenting any radiological sign of disease progression [52]. In the study of Castello and colleagues [53], they found that patients who experienced a PSA flare during ^223^Ra therapy had higher survival rates compared to patients with a prolonged PSA increase (“non-responders”). The outcomes of these patients were comparable to those who had an instantaneous PSA reduction. Moreover, experiencing a PSA flare was correlated to a lower tumor burden, as shown by ΔTLF10 and ΔFTV10 on 18F-fluoride PET/CT, where TLF10 is the total fluoride skeletal metastatic uptake, which is the product of mean SUV × SUVmax with threshold set at 10 (VOI10), while FTV10 is the total volume of fluoride bone metastases. These results showed how an increase in the first 2 months of treatment does not represent progression, so it is not necessary to discontinue radium. However, it is not clear if the rise in PSA blood levels is due to the lysis of PCs in bones due to the effects of the alpha particles or due to the bone remodeling. Some authors suggested that PSA is released into the bloodstream by tumor cells after lysis after the treatment transactivates mutant androgen receptors through its estramustine component and premedication with dexamethasone, or by the accelerated differentiation of prostate cancer stem precursors [54]. A study conducted on mice [55] demonstrated that ^223^RaCl_2_ localizes in the front of the bone growth plate in intact hind limbs and on the bone surface surrounding the tumor (independently if osteoblastic or osteolytic) in a metastasis model of osteoblastic and osteolytic bone PCa metastases. Also, the uptake was blood vessel density-dependent and was not influenced by the exposed bone surface or volume.

## 3. Genetics

Genome instability is another cancer-related aspect. Since cells have a complex DNA damage response (DDR), cells with DDR defects can be resistant to repair pathways [56]. DDRs are principally responsible for repairing two main types of DNA damage: single-strand breaks (SSBs) and double-strand breaks (DSBs) [57]. Defects in the DDR network could lead to hereditary and sporadic cancers. In particular, the loss of function of breast cancer 1 (*BRCA1*) and breast cancer 2 (*BRCA2*) is a predisposing factor for developing not only ovarian, pancreatic and breast cancers, but also prostate cancer. Their deficiency could be caused by germline or somatic gene mutations or from epigenetic silencing [58]. They affect the repair of DSBs through homologous recombination (HR), one of the three error-free systems involved in repairing DSBs [59], which consists of repairing the damaged DNA using an identical sequence [59]. These mutations expose the cells to alternative DSB repair systems such as non-homologous end-joining (NHEJ) and single-strand annealing (SSA), which are more error-prone. In similar cells, it was found that the inhibition of poly(adenosine diphosphate-ribose) polymerase enzymes 1 (PARP1) and 2 (PARP2), sentinels of DNA damage which activate SSB repair, (particularly base excision repair (BER)), induces apoptosis and cell cycle arrest through synthetic lethality [58]. Since *BRCA1* and *BRCA2* are key enzymes in the HR system, when they are mutated, the cells are said to be homologous recombination-deficient (HRD). This condition is associated with a higher sensitivity to poly(adenosine diphosphate-ribose) polymerase (PARP) inhibitors [60]. A promising approach for the evaluation of HRD is through searching for DNA “scars” due to DNA aberrations. Since HR permits error-free repair of double-strand breaks, in its absence, DNA collects these scars. Other genes are involved in HR, such as speckle-type POZ protein (SPOP), a tumor suppression gene often mutated in prostate cancer [61], which is a E3 ubiquitin ligase adaptor protein [62]. More aggressive prostate cancers are associated with loss-of-function alterations in the *BRCA1* and *BRCA2* genes and a major sensitivity to PARP inhibition has observed and analyzed in the study of de Bono and colleagues [63]. A longer progression-free survival (PFS) and better response were present in metastatic-castration resistant prostate cancer (mCRPC) patients harboring a *BRCA1*, *BRCA2* or ataxia telangiectasia mutated (*ATM*) mutation and with progression when they were treated with Olaparib, a PARP inhibitor. This cohort showed a 66% lower risk of death and progression compared to the control group that received enzalutamide or abiraterone. The previous or subsequent administration of chemotherapy did not influence the efficacy of the PARP inhibitor therapy. In the study of Sartor et al., a comparison between African American (AA) and Caucasian American (CA) PCa patients in terms of the frequency of pathogenic or likely pathogenic (P/LP) germinal variants was conducted. *BRCA* and non-*BRCA* germinal variants were lower in the AA group in general. Non-*BRCA* germline DNA repair mutations were less likely in the AA patients, while the risk of P/LP *BRCA* mutations was similar between the two groups [64]. Synthetic lethality is a lethal event that occurs when the co-existence of multiple gene mutations induces cellular death, while a single genetic event is bearable. This aspect is exploited by modern genetic therapy, in which the use of an inhibitor in cells with pre-existing mutations or overexpression can lead to cellular death [65]. This is also used in radium-223 therapy. It is well known that a combination of other therapies with radium provides benefit [66]. For example, the concomitant use of radium-223 with docetaxel demonstrated a good anti-tumor effect, prolonging the biochemical suppression and time to progression [67]. Radiotherapy is responsible for the release of danger-associated molecular patterns (DAMPs) that stimulate the immune response, resulting in DNA damage. Radium-223 itself targets osteoclasts, blocking their immunosuppressive properties. Similarly, the use of DNA damage repair treatments could potentially inhibit the repair of DSBs induced by radium-223 in the bone cancer microenvironment [68].

## 4. Combination Treatment with Radium-223

Given the mechanism of action of radium-223, several studies have tried to understand how radium can be combined with the currently used therapies (chemotherapy, immunotherapy and hormone therapy) in men with metastatic castration-resistant prostate cancer (mCRPC) to increase the effectiveness of this treatment [69].

The multicenter phase III ERA 223 trial assessed the efficacy and safety of the combination of ^223^Ra treatment to abiraterone plus prednisone or prednisolone in patients with asymptomatic or minimally symptomatic mCRPC [70]. Abiraterone acetate (abiraterone) is a selective steroidal inhibitor of cytochrome P450 c17 (CYP17), a key enzyme in the biosynthesis of testosterone and estrogen. Inhibition of CYP17 blocks the production of steroids in the testes, the adrenal glands and tumors, and thus limits the amount of hormones available to stimulate AR signaling [71,72]. In the ERA 223 trial, the overall survival between the group treated with abiraterone + radium versus patients treated with the placebo + abiraterone (33.3 versus 30.7 months; HR 1.195, 95% CI 0.950–1.505; *p* = 0.13) was not statistically different. However, patients who also received ^223^Ra had a higher incidence of fractures (29% versus 11%) [70]. Several reasons were proposed to explain the increased number of skeletal fractures in the combined therapy group compared with the control group: low-dose prednisone has a negative effect on bone metabolism by inhibiting osteoblasts and activating osteoclasts, leading to a decrease in bone formation [73]. In addition to the potentially harmful effects of glucocorticoids and possible hormonal changes in the bone microenvironment as a result of abiraterone use, the addition of ^223^Ra could contribute to further damage to bone health and lead to an increased incidence of skeletal fractures [73]. Interestingly, as shown in the ERA 223 trial, the use of bisphosphonates and denosumab may reduce the risk of fracture in patients treated with radium-223 [70]. Whether the increased fractures will be seen in patients receiving a combination of ^223^Ra and AR axis inhibitors other than abiraterone remains to be determined [74].

It has been shown that chemotherapy, when combined with radiotherapy, has a radiation-sensitizing effect through several mechanisms, including increased radiation-induced damage, reduced DNA repair, increased susceptibility of hypoxic cells to cytotoxic agents, inhibition of prosurvival pathways and reduced ability of cancer cells to repopulate after radiotherapy, leading to a cytotoxic effect [75]. On the other hand, data on the radiosensitizing effects of combination chemotherapy with alpha-particle emitters are currently unavailable [76]. The potential benefit of radiopharmaceuticals in combination with chemotherapy in patients with mCRPC has previously been demonstrated in clinical trials of chemotherapy in combination with beta-particle emitters [77]. For ^223^Ra, an exploratory efficacy analysis of a randomized phase I/II clinical trial showed that ^223^Ra (55 kBq/kg every 6 weeks; *n* = 36) plus docetaxel (60 mg/m^2^ every 3 weeks) may have better anti-tumor activity compared to docetaxel alone (75 mg/m^2^ every 3 weeks; *n* = 17) [78]: PSA levels (median time to progression 6.6/month vs. 4.8/month, P = 0.02), ALP levels (9/month vs. 7/month, P = 0.44) and markers of osteoblast activity were suppressed longer using the combination therapy [78,79]. The combination of ^223^Ra with immunotherapy, particularly the type targeting the generation and expansion of endogenous T cells, may be a beneficial approach [80]. In response to inflammatory stimuli, programmed death ligand 1 (PD-L1) is selectively expressed on many cancer cells and cells in the tumor microenvironment. T cell function is inhibited by the interaction between PD-L1 and its receptor, Programmed Death 1 (PD-1) [81,82]. Atezolizumab, a PD-L1 monoclonal antibody, binds to PD-L1 on tumor cells and prevents its interaction with PD-1 and B7.1 receptors on immunosuppressed T cells. This leads to T cell activation and tumor cell death through specific immune responses [83]. Preclinical studies suggest that activation of the PD-L1-PD-1 pathway may reduce radiation-induced immune responses. Therefore, antibodies against PD-L1 were tested in a mouse model. An improvement in the activation of the immune response by ionizing radiation was observed, through a mechanism that was dependent on cytotoxic T cells [84]. There are ongoing clinical trials evaluating the effectiveness of ^223^Ra in combination with the PD-L1 inhibitor atezolizumab in patients with mCRPC who have experienced progression after treatment with an androgen pathway inhibitor, and ^223^Ra in combination with pembrolizumab in patients with mCRPC and bone metastases [85]. The DNA damage response maintains cellular integrity and homeostasis. The cellular detection of DNA damage activates DNA damage response pathways, leading to cell cycle arrest and the induction of DNA repair or cell death [86]. In 12–30% of men with advanced CRPC, genomic DNA repair defects have been identified [87,88,89]. Some of these tumors with genetic alterations are sensitive to DNA-damaging anti-cancer drugs, including platinum-based chemotherapy, and to inhibitors of the DNA damage response proteins, including PARP [90]. PARP is involved in repairing endogenous SSB breaks using the Base excision repair (BER) pathway. PARP inhibitors suppress PARP and thus the repair of SSBs through the BER pathway. Unrepaired SSBs induce apoptosis and lead to cell death [91]. As ^223^Ra is able to induce double-strand DNA breaks, inhibition of DNA damage repair pathways could increase its effectiveness [92]. Therefore, the combination of ^223^Ra with PARP inhibitors may be useful, as a PARP inhibitor could further reduce the ability to repair double-strand breaks in cells irradiated with ^223^Ra, perhaps even in patients whose tumors do not show alterations in DNA repair genes. Trials have evaluated the PARP inhibitor niraparib in combination with ^223^Ra in patients with mCRPC without DNA repair gene alterations and established the safety of this combination, and a randomized phase I/II study is planned to evaluate the combination of olaparib and ^223^Ra [93,94]. A 2018 study investigated whether homolog-recombination gene mutations correlate with ^223^Ra efficacy in patients with mCRPC and bone metastases: the ALP response (80% vs. 39%, P = 0.04), progression-free survival (median 10.4 months vs. 5.8 months, HR = 6.4, P = 0.005) and overall survival (median 36.9 months vs. 19.0 months, HR = 3.3, P = 0.11) were longer in patients treated with ^223^ Ra than in those not treated with radium. Unfortunately, the correlation between homologous recombination deficiency status and response to ^223^Ra requires further validation, as the patient population was small (*n* = 28) [95]. The main findings concerning the combination of ^223^Ra therapy with other therapeutic regimens are summarized in Table 1.

## 5. Physiopathology of the Cancer Microenvironment

**Figure 1:**
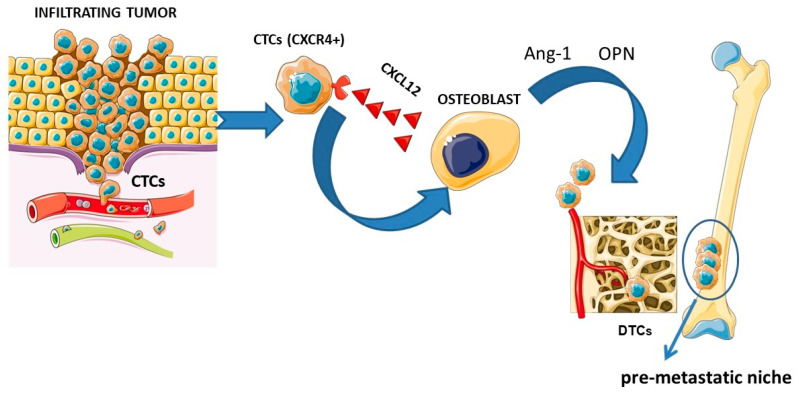
Schematic representation of the “seed and soil” theory. From right to left: primary tumor with tumor cells detaching, migrating through the vascular system, and attracting of circulating tumor cells (CTCs) through the interaction between the CXCR4 receptor and the CXCL12 ligand released by osteoblasts and stimulation by angiopoietin (Ang-1) and osteopontin (OPN), prompting the CTCs to enter the bone (the CTCs become disseminating tumor cells (DTCs)), leading to the formation of a “pre-metastatic niche”. Figure was drawn using pictures from servier medical art. Servier medical art by servier is licensed under a creative CommonsAttribution 3.0 unported license (https://creativecommons.Org/licenses/by/3.0/ accessed on 8 February 2025).

To better understand the possible effects of ^223^Ra on the microenvironment, the physiopathology of the cancer microenvironment should be analyzed in detail. As previously mentioned, radium-223 actively binds the sites of active osteoblasts; thus, it is pivotal to focus on the principal molecular pathways which influence the formation of bone metastases. Cancer metastases represent the main cause of cancer-related mortality, particularly in prostate and breast cancers (more than 70% of these malignancies develop metastases) [96]. Bone metastases are characterized by a dysregulation of bone metabolism, increased bone resorption or abnormal bone formation. The metastases are typically classified in osteoblastic, which are characterized by the predominance of newly formed bone tissue and a modest osteolytic component (e.g., prostate cancer), and in osteolytic, characterized by significant bone resorption (e.g., in breast cancer), cancers [97]. In fact, both osteoclasts and osteoblasts, as well as their progenitors, are stimulated to change by the invading tumor cells. Therefore, tumor growth in bone can result in an osteolytic (bone resorbing), osteoblastic (bone creating) or mixed bone lesion, depending on the types of activated cells [68]. One key step in metastasis is the entry of circulating tumor cells (CTCs) into secondary or distant organs, which become disseminated tumor cells (DTCs) for subsequent metastasis. DTCs have stem-cell like properties such as proliferative activity, resistance to apoptosis, self-renewing activity and a differentiation ability [98]. Bone metastases derive from DTCs that reach the bone tissue and progress into metastatic growths through activation during bone remodeling. Bone remodeling itself increases the number of PCa metastases in mice bone tissue [99,100] and the number is influenced by the increased activity of DTCs rather than the number of cancer cells homing to the bone tissue. DTCs self-amplify and produce factors responsible for the activation of osteoclasts, which create new spaces for the tumor and osteoblasts which produce new bone. Other growth factors are then released to permit tumor growth [68]. Primary tumors can “prepare” the local microenvironment of distant organs for tumor cell colonization even before their arrival. The “seed and soil” hypothesis by S. Paget has been instrumental in our understanding of tumor metastasis and provides an explanation for the organotropism of metastasis: pro-metastatic tumor cells (the “seed”) colonize in specific organ sites (the “soil”) where the microenvironment is favorable for metastasis. Increasing evidence shows that the primary tumor can promote metastasis by inducing the formation of a supportive microenvironment in a secondary organ site, termed the pre-metastatic niche. The pre-metastatic niche is the fertile ground (seed) in which the metastatic cancer cell is located (soil); in fact, through a series of molecular and cellular changes, it becomes a supporting tissue allowing for the establishment of cancer cells and consequently the development of distant metastases [96]. The metastasis process begins with the detachment of malignant cells from the primary tumor and the migration of these cells into the nearby vasculature. A complex of cell adhesion molecules, including selectins and cadherins, maintains cell adhesion in the normal prostate gland. The expression of different molecules is altered early in the migration process by prostate cancer cells, leading to decreased cellular adhesion [101]. The initial attraction of detached cells to distal sites is primarily controlled by integrins and chemokines produced by the bone marrow and stromal cells once the cells intravasate [102]. The CXCL12 receptor, C-X-C chemokine receptor 4 (CXCR4), is present on osteoclast precursors and governs the migration of blood cells into bone [103]. Like hematopoietic stem cells (HSC) precursors, cancer cells express CXCR4 and are therefore drawn to the bone microenvironment [104]. In addition to producing large amounts of CXCL12, osteoblasts also release anchoring molecules like angiopoietin (Ang-1) and osteopontin (OPN) that stimulate tumor cells to enter the bone microenvironment. In this regard, high levels of OPN have been found metastatic cells and stromal tissue in both prostate and breast cancers [105]. The “seed and soil” hypothesis is schematized in Figure 1.

Annexin II is another molecule involved in the homing of HSCs to the niche: it binds to its receptor on the surface of osteoblasts, regulating bone homing in a similar way to the interaction of CXCL12/CXCR4 [106].

When the tumor cells reach the niche, they experience growth arrest, resulting in ‘’quiescence’’ [107,108], that is the ability to engage in a reversible state of cell cycle arrest [101]. Ewing surmised that the vascular system anatomy can influence the metastasis process. This is the reason why the metastases are typically found in more vascular organs (for instance, in the lungs, liver, skeleton, brain and adrenal glands) [109]. In case of bone tissue, osteomimicry, which is the ability to acquire a bone-cell phenotype similar to osteoblasts, is one of the factors that increases the possibility that tumor cells survive and proliferate in bone tissue [96,110]. Interestingly, the time of bone recurrence is equal to total skeletal renewal time, highlighting the importance of bone remodeling in metastasis, and it is also sustained by hormones such as parathyroid hormone (PTH), which induces the perfect conditions for hematopoietic stem cell proliferation [111] and for dormant tumor cells [68,112]. Bone tissue is a dynamic system subject to a constant mechanism of formation and resorption, the basis of which is the crucial role played by a system consisting of receptor activator RANKL, its receptor activator of NF-kappaB (RANK) and its decoy receptor osteoprotegerin (OPG), which regulate all aspects of osteoclast function, from differentiation to apoptosis [113,114].

RANK is a transmembrane receptor expressed on active precursors and osteoclasts; RANK-L is the ligand of RANK and is present on osteoblasts, T cells and bone marrow stromal cells. This ligand–receptor binding favors the formation, activation and survival of osteoclasts. OPG, RANK’s decoy receptor, facilitates the apoptosis of the activated osteoclasts and inhibits the differentiation of osteoclasts. For this reason, the balance between OPG and RANK-L influences the differentiation, activation and survival of osteoclasts [115,116,117]. In patients with bone metastases, a breakdown of the RANK pathway is observed, which causes an imbalance in the processes of resorption and bone formation as a result of a disequilibrium between RANK-L and OPG: when cancer cells reach bone, the release of growth factors such as fibroblast growth factors (FGFs), endothelin-1 (ET-1), insulin-like growth factor-1 (IGF-1), platelet-derived growth factor (PDGF), transforming growth factor β (TGF-beta) and wingless-related integration site (WNT) is stimulated [118,119]. These factors induce osteoblasts to produce and release RANK-L and activate osteoclasts, which favor bone resorption. Moreover, there are growth factors released by the osteoclasts themselves (for instance, TGF- beta and FGF) that favor further bone resorption and the tumor growth, thus promoting a vicious cycle of tumor spread and bone resorption [120]. In osteoblastic PCa lesions, prostate-specific antigen (PSA) plays a crucial role, stimulating osteoblasts and releasing osteoblast-inactivating parathyroid hormone-related protein (PTHrP) [121,122]. WNT proteins are a family of glycoproteins involved in the development of metastases: their activity is closely related to the levels of beta-catenin. In fact, when WNT binds to its receptors (Frizzled and LRP-5/6), there is an increase in the intracellular levels of beta-catenin which, after it translocates to the nucleus, binds to several transcription factors, modifying the expression of different genes including those coding for some components of the cellular bone matrix, proteins involved in the cell cycle and oncogenes capable of inducing tumor development, like C-myc. In addition, WNT promotes osteoblastogenesis by increasing the expression of OPG on osteoblasts, resulting in increased differentiation, proliferation, survival and activity of osteoblasts and the development of osteoblastic metastases [120]. According to the evidence, WNT released from metastatic prostate cancer cells can stimulate osteoblasts and promote tumor growth, while Dickkopf-1 (DKK1), which inhibits WNT signaling, can lead to osteolysis, particularly in the early stages of cancer development. DKK1 expression is elevated in early-stage prostate cancer, with a decrease in DKK1 levels observed in advanced bone metastases. It is suggested that tumor establishment necessitates the initial upregulation of DKK1, while a reduction in DKK1 during bone metastasis can lead to an increase in WNT expression, resulting in osteoblastic metastases that are traditionally associated with prostate cancer [123,124]. Bone morphogenetic protein (BMP), TGF-β, IGF, PDGF, vascular endothelial growth factor (VEGF) and endothelin-1 (ET-1) are other paracrine prostatic factors that regulate the proliferation and differentiation of osteoblasts [125]. Numerous members of the TGF-β family have also been found to stimulate bone formation. Serum TGF-β concentrations in patients with prostate cancer were higher in patients with bone metastases than in non-bone-metastasis patients [126]. Both adipocytes and osteoblasts share the same precursor, known as mesenchymal stem cells (MSCs). The regulation of bone mass and homeostasis depends on the presence of adipogenic (e.g., c-receptor activated peroxisome proliferator (PAPARg)) or osteogenic factors ((e.g., runt-related transcription factor 2 (Runx2) and core-binding factor alpha 1 (Cbfa1)) that may be present in the bone microenvironment; the behavior of tumor cells has been shown to be influenced by the presence of adipocytes and adipocyte-associated factors. Cancer cells, unlike normal epithelial cells, are able to store and use lipids in order to gain a growth advantage. Additionally, tumor proliferation and tumor survival are influenced by the secretion of adipokines and cytokines produced by adipocytes and inflammatory cells [127]. Herroon et al. demonstrated that the presence of factors related to adipocytes can lead to a more advanced phenotype. In particular, an increase in invasion was observed in prostate cancer cells exposed to fatty acid-binding protein 4 (FABP4) [128]. Furthermore, marrow adipocytes can secrete ligand 1 (CXCL1) and ligand 2 (CXCL2), C-X-C chemokines, which can activate osteoclasts, thus supporting the vicious circle of cancer-induced bone disease [129]. Bone marrow MSCs can generate different cell types including osteoblasts, adipocytes, chondrocytes and fibroblasts [130]. It is well known that MSCs are recruited to the primary tumor site to promote tumor progression and metastasis. This is due to the presence of C-X-C (CXCR6) chemokine receptor 6 in prostate cancer. CXCR6 signaling supports the recruitment, conversion and activation of MSCs into cancer-associated fibroblasts (CAFs) that secrete CXCL12 [131]. CAFs are recognized as crucial players in the growth and metastasis of primary tumors, and their crucial role in tumor growth in the bone environment is beginning to be recognized [132]. The contribution of some proteins of the bone matrix in the metastatic process is another important aspect. These proteins include Osteocalcin (OC) and sialoproteins (BSPs), such as osteopontin (OPN). Both appear to be overexpressed in prostate cancer cells, giving them the ability to spread and settle in bone tissue, following the binding to integrin matrix receptors. Moreover, the recruitment of osteoblasts and osteoclasts is facilitated by OC, which contributes to bone turnover dynamics and the onset and development of bone metastases [96]. Chronic inflammation also contributes to the development of tumors and metastases through the activation of different signal pathways. Insufficient Toll-like receptor 4 (TLR-4) signaling activation in tumor cells can result in inflammatory responses, which can lead to resistance to tumor cell death and more active proliferation and invasion. Furthermore, TLR-4 and other innate sensors in immune cells that are not activated properly can lead to unresolved inflammation, which can lead to tumor progression and metastasis [133]. Toll-like receptor 5 (TLR-5) signaling and hypoxia within the pre-metastatic niche are responsible for the upregulation of interleukin-6 (IL-6) and tumor-promoting inflammation [134]. The seeding process, survival and proliferation of tumor cells can be assisted by the establishment of an inflammatory milieu at a secondary site, either before or at the same time as the arrival of CTCs [96]. According to recent data and research, lymphangiogenesis, the generation of new lymphatic vessels from pre-existing lymphatics or lymphatic endothelial progenitors, is a factor that promotes the migration of tumor cells [135].

The pre-metastatic niche may increase angiogenesis and vascular permeability in the development of metastases; this is due to the fact that the recruited TIE2+ monocytes and endothelial progenitor cells in the pre-metastatic niche create a proangiogenic microenvironment with high levels of VEGFs and other proangiogenic factors, which stimulate the angiogenesis and the consequent formation of metastases [136]. Lymphangiogenesis within the pre-metastatic niche is a factor that favors tumor metastases, and the lymphatic vessels (LVs) can become the route through which the tumor spreads [137]. According to clinical data, tumor-derived VEGF-A and VEGF-D cause pro-metastatic lymphangiogenesis in regional lymph nodes (LNs) and are associated with higher LN metastasis [135]. The lymphatic endothelial cells (LECs), a component of LVs within the pre-metastatic niche, can be conditioned by tumor-secreted IL-6 to express CCL5 and VEGF, which facilitate C-C chemokine receptor type 5-positive (CCR5+) tumor cell recruitment, extravasation and colonization into the niche [138]. Dendritic cells (DCs) may induce lymphangiogenesis for pre-metastatic niche formation during LN metastasis through cyclooxygenase-2/EP3-dependent induction of stromal cell-derived factor 1 (SDF-1), a chemokine involved in cell migration and known to be able to promote angiogenesis [139]. Moreover, lymphangiogenesis in the pre-metastatic niche (tumor-draining LNs) is important in initial tumor invasion, distal metastasis and immune unresponsiveness by modifying the host immunological responses through LEC-secreted factors, and through recruiting immature DCs and naive T cells [140]. In addition, the importance of lymphangiogenesis in tumor metastases can be accentuated by the permeable nature and slow lymphatic flow of LVs [96].

The dynamics of lymphogenic metastasis, especially the role of LVs in the pre-metastatic niche in metastasis, are not very clear. Immunosurveillance has the potential to lead to abortive tumor progression, including tumor metastasis. To overcome immunological elimination, tumors and their metastatic derivatives must develop strategies, such as establishing an immunosuppressive pre-metastatic niche. Anti-tumor immune responses may be suppressed by regulatory or immunosuppressive cells, like myeloid-derived suppressor cells (MDSCs), macrophages and Treg cells, within the pre-metastatic niche [133,141]. In particular, MDSCs that have accumulated in the pre-metastatic niche also inhibit anti-tumor T cells through arginase 1 (ARG1), resulting in a decrease in CD3 on T cells, cyclin D3 and cyclin-dependent kinase-4 (CDK4) on lymphocytes and reactive oxygen species (ROS) production, through mechanisms involving the inhibition of Janus kinase 3 (JAK3) and signal transducer and activator of transcription 5 (STAT5), a decrease in major histocompatibility complex 2 (MHC II) molecules and induction of apoptosis due to the suppression of the functions of T cells [142]. In addition, peroxynitrites, the product of the chemical reaction between nitric oxide (NO) and the superoxide anion, are present at sites where MDSCs and inflammatory cells accumulate and they can induce the nitroxylation of tyrosine residues on TCRs (T cell receptors) on T cells, causing alterations to the binding sites for the antigen and therefore insensitivity to antigen stimulation [143,144]. All of these molecular mechanisms contribute and seem to be entwined with some of radium’s actions, as stated in Section 2. The impact of ^223^Ra on the immune response to cancer and tumor cell phenotypes and studies on its localization in bones highlight that the action of ^223^Ra transcends its well-known categorization as a calcium analogue. There are no detailed explanations about the complex molecular action mechanism of radium in the bone microenvironment. Hence, there is an urgent need to conduct further studies to possibly better understand the clinical application of this promising (and maybe not yet old-fashioned) isotope.

## 6. Conclusions

The tumoral microenvironment is a complex setting in which complex interactions between cells and molecules are occurring. Radium-223, as an alpha-emitter, is capable of inducing DSD breaks. It is interesting to potentiate this effect in patients with genetic instability but also in combination with therapies which inhibit DNA repair, modulate immune responses, or control tumoral growth. Currently, there are different ongoing trials and further studies are needed. Different biomarkers have been proposed for the evaluation of therapeutic responses, like ALP levels which are precise and can be confidently used in clinical practice. PSA levels, conversely, are not a precise marker since they are affected by the flare phenomenon and its biological role is not clear. Given the breadth of this topic, understanding its roles in the tumor microenvironment represents a priority to better comprehend how to effectively exploit this alpha-emitter and its captivating action mechanism.

## Figures and Tables

**Table 1:** Main findings regarding combination treatments involving radium-223.

Study/Combination	Key Findings
ERA-223 Trial (Radium-223 + Abiraterone + Prednisone/Prednisolone)	The overall survival was not statistically different between the combination therapy and control groups (33.3 months vs. 30.7 months; HR 1.195, P = 0.13). A higher incidence of fractures was observed in the combination group (29% vs. 11%). The proposed causes included the following: effects of prednisone, hormonal changes from abiraterone, and radium-223’s impact on bone health. The use of bisphosphonates or denosumab may prevent fractures.
Radium-223 + Docetaxel (Randomized Phase I/II Trial)	The combination had better anti-tumor activity compared to docetaxel alone, with longer suppression of PSA progression (6.6 months vs. 4.8 months; P = 0.02) and markers of osteoblast activity.
Radium-223 + Immunotherapy (PD-L1 Inhibitors: Atezolizumab, Pembrolizumab)	There are ongoing clinical trials evaluating the effectiveness of this combination in mCRPC patients with progression after treatment with androgen pathway inhibitors. Preclinical studies suggest that PD-L1 inhibitors improve radiation-induced immune responses. The mechanism involves the activation of cytotoxic T cells.
Radium-223 + PARP Inhibitors (Niraparib, Olaparib)	PARP inhibitors may increase radium-223 effectiveness by reducing the DNA repair of radium-induced double-strand breaks. The initial trials established safety; further randomized studies are planned. The preliminary findings included improvements in the alkaline phosphatase (ALP) response, progression-free survival and overall survival in small patient groups.
Mechanism Synergy	In chemotherapy, the radiation-sensitizing effects include increased DNA damage and reduced repair. In immunotherapy, PD-L1 blockade enhances the immune response against tumors. Using DNA repair inhibitors, the synergistic effects are due to enhanced DNA damage in cancer cells.
Safety and Considerations	Combination therapies pose increased risks (e.g., fractures with abiraterone and radium-223). Patient selection based on biomarkers like ALP levels may improve outcomes. Further studies are required to confirm the benefits and mitigate the adverse effects.

PSA: prostate-specific antigen; HR: hazard ratio; P: probability; mCRPC: metastatic castration-resistant prostate cancer; PD-L1: programmed death ligand 1; ALP: alkaline phosphatase; PARP: poly(ADP-ribose) polymerase.

## Data Availability

Data available in a publicly accessible repository.
